# Ultrasound Assessment of Changes in Nails in Psoriasis and Psoriatic Arthritis

**DOI:** 10.1155/2018/8251097

**Published:** 2018-09-09

**Authors:** Magdalena Krajewska-Włodarczyk, Agnieszka Owczarczyk-Saczonek, Waldemar Placek, Maja Wojtkiewicz, Andrzej Wiktorowicz, Joanna Wojtkiewicz

**Affiliations:** ^1^Department of Rheumatology, Municipal Hospital in Olsztyn, 10-900 Olsztyn, Poland; ^2^Department of Internal Medicine, School of Medicine, Collegium Medicum, University of Warmia and Mazury, 10-900 Olsztyn, Poland; ^3^Department of Pathophysiology, School of Medicine, Collegium Medicum, University of Warmia and Mazury, 10-900 Olsztyn, Poland; ^4^Department of Dermatology, Sexually Transmitted Diseases and Clinical Immunology, School of Medicine, Collegium Medicum, University of Warmia and Mazury, 10-900 Olsztyn, Poland; ^5^Faculty of Earth Sciences, Department of Geomatics and Cartography Nicolaus Copernicus University, Torun, Poland; ^6^DRAMIŃSKI S.A. Ultrasound Scanners, Olsztyn, Poland; ^7^Laboratory for Regenerative Medicine, School of Medicine, Collegium Medicum, University of Warmia and Mazury, 10-900 Olsztyn, Poland

## Abstract

**Aim of the Study:**

The aim of the study was to conduct an ultrasound (US) assessment of changes in fingernails in psoriatic patients with nail involvement.

**Material:**

A total of 69 patients with psoriatic changes in nails participated in the study, including 38 patients with psoriasis (Ps) and 31 with psoriatic arthritis (PsA) and 30 people in the control group. A total of 988 nails were examined.

**Results:**

The thickness of the nail plate, nail bed, and matrix as shown in an ultrasound examination increased with the mNAPSI index (r=0.328, p=0.021; r=0.219, p=0.036; and r=0.422, p=0.011, respectively). The thickness of nail plate, bed, and matrix in patients with onycholysis and hyperkeratosis-type changes (concomitant or present separately) was significantly greater than when only pitting-type changes occurred (p=0.007, p=0.035, and p=0.023, respectively). An examination of nails with only pitting-type changes showed an increase in the matrix thickness compared to the control group (p=0.018). The focal hyperechoic involvement of the dorsal plate (80%) was the change most often observed in an US examination in Ps patients, whereas loosening of the borders of the ventral plate was most often observed in PsA patients. The thickness of nail bed in PsA patients increased with the duration of arthritis (r=0.399, p=0.022) and was correlated with the number of swollen digits (r=0.278, p=0.041).

**Conclusions:**

The findings of this study may indicate an association of an inflammation in the nail bed with PsA development. Apart from a direct assessment of the described morphological changes of nails, a US examination could prove useful in an assessment of intensity of a local inflammation as a prognostic factor for PsA development.

## 1. Introduction

Psoriatic changes in nails are among the clinical manifestations of psoriasis (Ps). Apart from a characteristic picture of a local condition, such changes are often accompanied by pain and functional restriction of hand mobility. Psoriatic changes in nails are a known risk factor for development of psoriatic arthritis (PsA) [1], which particularly affects adjacent distal interphalangeal joints, digital extensor tendon, and sites of their attachment [2–5]. Nail involvement in psoriasis is observed in 10-55% of psoriatic patients, but the risk of occurrence of such changes during a patient's lifetime may be as high as 80-90% [6]. An assessment of psoriatic changes in nails in clinical practice is based on a physical examination and clinical assessment indices, such as nail psoriasis severity index (NAPSI) and modified NAPSI (mNAPSI) [7, 8]. Useful imaging techniques include ultrasound (US) examination [9] and magnetic resonance imaging (MRI), so far being the main methods in imaging of articular changes [10] and optical coherence tomography [11]. Being a noninvasive and a relatively cheap method, US seems to be highly promising for use in assessment of intensity, progression, and treatment outcome of changes in nails and adjacent structure.

## 2. Aim of the Study

The aim of the study was to conduct an ultrasound assessment of changes in fingernails in psoriatic patients with nail involvement and an assessment of an association of the morphological changes as assessed by ultrasound with selected clinical factors.

## 3. Materials and Methods

A total of 99 patients participated in the study, including 69 successively registered patients with psoriasis (38 with psoriasis and 31 with psoriatic arthritis) with psoriatic changes in at least one fingernail, treated at the Dermatology Clinic and at the Rheumatology Clinic of the Municipal Hospital in Olsztyn and at the Clinic of Dermatology, STDs, and Clinical Immunology at the University of Warmia and Mazury. Thirty people without psoriasis or psoriatic arthritis were the control group. Psoriatic arthritis was diagnosed based on CASPAR criteria [12]. The patients' age ranged from 33 to 64 years.

All the patients were examined by an experienced dermatologist. The macroscopic progression of psoriatic changes in nails with pitting, hyperkeratosis, and/or onycholysis was assessed by mNAPSI. The intensity of psoriatic changes in the skin was assessed with the PASI (Psoriasis Area and Severity Index) [13].

The activity of psoriatic arthritis was assessed by the Disease Activity Score calculated for 28 joints (DAS 28) [14] and the number of tender (tender joint count, TJC) and swollen (swollen joint count, SJC) joints, calculated for 68 and 66 joints, respectively.

A US examination of nails and distal interphalangeal (DIP) joint extensor tendons was conducted in all the patients and people in the control group. The examination was conducted by a rheumatologist experienced in ultrasound examinations of the skeletal and muscular system. Morphological changes were examined with DermaMed equipment and software (Dramiński, Olsztyn, Poland) with a linear head with frequency ranging from 12 to 48 MHz. All the nail examinations (988 nails were examined; 2 nails were excluded because of a previous injury) were conducted at 24 MHz. An assessment of intensified blood supply, corresponding to intensification of inflammation, was made with a Mindray M5 (Mindray, Guangdong, China) apparatus with the Power Doppler (PD) technique. An assessment of the nails, extensor tendons, and DIP joints was made by placing the head on the dorsal side. In order to avoid pressure on surface tissues, an appropriate amount of gel without gel pads was used. In order to avoid artefacts, the intensified flow, visible in the PD technique, was confirmed by pulsed wave Doppler spectrum. The nail thickness was measured as the maximum distance between the dorsal and ventral nail plates. The nail bed thickness was measured as the distance between the ventral plate and the bone margin of the distal phalanx. The nail matrix thickness was measured at the proximal end of the nail bed.

According to the classification proposed by Wortsman et al. [15], morphological changes in nails in US examinations were described as focal hyperechoic involvement of the dorsal plate (type I), loosening of the borders of the ventral plate (type II), wavy plates (type III), and loss of definition of both plates (type IV).

Entheses were assessed in accordance with OMERACT (Outcome Measures in Rheumatology) [16] recommendations in a US examination, in the scale of greyness, at the place where an extensor tendon is attached to the distal phalanx of the DIP joint. Loss of normal fibrillary architecture, thickened tendon, or enthesophytes at its bony insertion and bony changes including erosions were regarded as enthesopathies. The tendon thickness was measured at the place where it is attached to the distal phalanx.

Inflammatory markers were measured with two standard laboratory parameters: erythrocyte sedimentation rate assessed using BD Vacutainer Sedi-15 equipment (BD, Franklin Lakes, NJ, USA) and concentration of C-reactive protein measured with a standard immunoturbidimetric method using a COBAS 6000 INTEGRA apparatus (Roche Diagnostics, Mannheim, Germany).

Patients with changes in nails other than those caused by psoriasis were excluded from the study. Manual labourers were also excluded from the study in order to eliminate from the assessment any effect of injuries on nail assessment.

All patients gave their written consent to participation in the study. The experiment was approved by the Bioethics Committee of the Warmia and Mazury Chamber of Physicians (OIL 625/16/Bioet; 21.12.2016).

## 4. Statistical Analysis

StatSoft program, Inc. STATISTICA, version 12.5 (StatSoft, Tulsa, OK, USA), was used for calculations. The results were presented as an arithmetic mean and standard deviation. The Mann–Whitney* U* test and the Kruskal-Wallis test were used for comparative analysis between the groups. The presence of the relationship between quantitative features was tested using Pearson's correlation coefficient for parameters consistent with normal distribution and Spearman's correlation coefficient for noncompliance with normal distribution. The statistical level of significance was p <0.05.

## 5. Results

A total of 99 patients aged 33-64 years participated in the study, including 69 patients with nail psoriasis (38 with psoriasis without arthritis and 31 with psoriatic arthritis) and 30 people in the control group. There was no difference between the groups regarding the age or sex. The duration of psoriasis in both groups of patients did not differ significantly. Greater intensity of dermal changes was observed in patients with psoriasis without arthritis compared to PsA patients. No differences were observed in both groups regarding the intensity of changes in nails as assessed with the mNAPSI. Significantly higher inflammation indices were observed in patients with arthritis than those with psoriasis (Table 1.).

**Figure 1:**
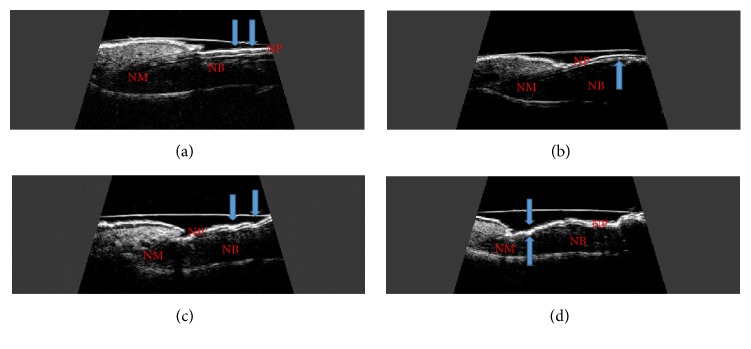
Longitudinal scan of psoriatic nail. (a) Focal hyperechoic involvement of the dorsal plate. (b) Loosening of the borders of the ventral plate. (c) Wavy plates. (d) Loss of definition of both plates. NP: nail plate; NB: nail bed; NM: nail matrix.

A total of 988 nails were examined: 380 nails in patients with psoriasis without arthritis, 310 nails in patients with PsA, and 298 nails in people in the control group. Psoriatic changes in patients with Ps and PsA were present in 248 (65%) and 168 (54%) nails, respectively. The intensity of dermal changes in the studied patients was not associated with the nail plate, bed, or matrix. The thickness of the nail plate, nail bed, and matrix increased with the mNAPSI index in all the patients (r=0.328, p=0.021; r=0.219, p=0.036; and r=0.422, p=0.011, respectively). The nail plate thickness was correlated with the duration of psoriasis in all the patients (r=0.275, p=0.029) regardless of the presence of macroscopic changes in nails. The US examination of the affected nails revealed an increased thickness of the nail plate, bed, and matrix in both groups of patients compared to the control group (Table 2). An examination of digits with no nail involvement in Ps and PsA patients revealed increased thickness of the nail bed compared to the control group (Table 3.). As it was difficult to group the patients in regard to the clinical symptoms of nail psoriasis because of frequent occurrence of concomitant changes, the patients were divided into groups with only pitting-type changes and those with other symptoms, including onycholysis and hyperkeratosis. The nail plate, bed, and matrix thickness in patients with onycholysis and hyperkeratosis-type changes (concomitant or present separately) were significantly greater than when only pitting-type changes occurred (p=0.007, p=0.035, and p=0.023, respectively). An examination of nails with only pitting-type changes showed an increase in the matrix thickness compared to the control group (p=0.018). The nails with psoriatic changes were assessed in a US examination in regard to their morphology, in accordance with the classification proposed by Wortsman et al. (Figure 1). Focal hyperechoic involvement of the dorsal plate (type I), loosening of the borders of the ventral plate (type II), and wavy plates (type III) in patients with psoriasis were observed in 86.5%, 10%, and 3.5% of the nails under examination, respectively. No loss of definition of both plates (type IV) was observed. Types I, II, III, and IV changes were present, respectively, in 16%, 77%, 5%, and 2% of the patients with PsA (Table 4). Moreover, the nail bed thickness in PsA patients increased with the duration of arthritis (r=0.399, p=0.022) and was correlated with the number of swollen digits (r=0.278, p=0.041). However, no correlation was found between the bed thickness and the number of tender joints or disease activity as measured by the DAS28 index.

Increased vascularisation as assessed by the PD method in the nail matrix area in Ps and PsA patients was observed with a similar frequency. An increased PD signal in the nail matrix in Ps was observed in 75/248 (30%) digits with psoriatic nails and in 17/132 (13%) digits with no clinical changes in the nails (p=0.023). Increased vascularisation in the matrix area in digits of patients with arthritis was observed in 57/168 (34%) of digits with changes in nails and in 18/142 (12%) digits with no changes (p=0.019). A PD signal of increased intensity was observed more frequently in PsA patients, in 101/310 (32.5%) digits, than in Ps – in 91/380 (24%) digits (p=0.031). Increased vascularisation in the nail bed in Ps patients was observed in 78/248 (31%) and 13/132 (10%) digits with and without nail involvement, respectively (p= 0.007). Increased vascularisation in the nail bed in PsA patients was observed in 75/168 (45%) and 26/142 (18%) digits with and without nail involvement, respectively (p= 0.027). An intensified PD signal in the nail bed and matrix in the control group was observed in 6/298 (2%) and 8/298 (2.6%) of the examined digits.

The thickness of digital extensor tendons in both patients groups was significantly greater than in the control group (Table 2). On the greyness scale, in the US examination of entheses of digital extensors with clinical involvement of nails and with no changes in nails in the groups of patients with Ps and PsA, changes of the type of loss of normal fibrillary architecture, enthesophytes, and bony changes including erosions were observed more frequently in DIP joints of digits with nail involvement. In patients with Ps, loss of normal fibrillary architecture, enthesophytes, and bony changes were observed in 75/248 (30%) versus 25/132 (19%), 36/248 (14.5%) versus 12/132 (9%), and 17/248 (7%) versus 4/132 (3%) DIP joints of digits with and without changes in nails, respectively. In patients with Ps, enthesopathies were present more frequently in digits with nail involvement than in those in which nails were not affected: (79/248 (31%) versus 34/130 (26%), respectively, p=0.047). In patients with PsA, loss of normal fibrillary architecture, enthesophytes, and bony changes were observed in 87/168 (52%) versus 63/142 (44%), 46/168 (27%) versus 24/142 (17%), and 34/168 (20%) versus 16/142 (11%) DIP joints of the digits under examination with and without involvement of the nails. In patients with PsA, enthesopathies were present in 114/168 (68%) digits with nail involvement and in 80/142 (56%) digits with no changes in the nails; the differences were not statistically significant. No erosions were observed in the tendon attachment sites under study in the control group. Loss of normal fibrillary architecture and enthesophytes were observed in this group in 17/298 (6%) and 14/298 (5%) digits, respectively.

## 6. Discussion

Psoriatic changes in nails are a frequent manifestation of psoriasis and a known risk factor for the development of arthritis [4, 17].

Significantly thicker nail plates, bed, and matrix in nails with psoriatic changes were observed in the Ps and PsA patients compared to the control group. The thickness of the nail plates, bed, and matrix was correlated with the mNAPSI index, just like in the study by Gisondi et al. [18]. The thickness of the nail bed in the PsA patients in the current study increased with the duration of arthritis and was correlated with the number of swollen digits, but not with the number of tender joints or disease activity as measured by DAS28. Increased intensity of PD signals in the nail bed and matrix was observed more frequently in Ps and PsA patients than in the control group. Increased thickness of the nail bed was observed in nails without psoriatic changes in both groups of patients. It is also here that we observed increased PD flows in an examination of the nail bed and matrix compared to the control group. These changes may suggest that inflammation was developing within the nail and they may have preceded the emergence of clinical changes in nails. An increase in the nail plate and matrix thickness in patients with and without psoriatic changes in nails was reported by Aydin et al. [19]. Gisondi et al. found the nail plate and bed to be greater in Ps patients regardless of the existing nail changes [18]. In another study, Sandobal et al. observed greater bed thickness and increased PD signal in the nail bed in Ps and PsA patients [20]. Ally Essayed et al. assessed the thickness of the nail plate, bed, and matrix in the nails of the thumb and the second hand digit. An increase in the nail plate thickness in the thumb and the index finger over 0.63 mm and 0.61 mm, respectively, as a manifestation of nail psoriasis had a sensitivity of 72% and 60% and specificity of 70% and 88%, respectively. The thickness of the thumb nail bed of over 1.85 mm (sensitivity of 64% and specificity of 72%) had a similar diagnostic value in this examination. The thickness of the index finger nail bed of over 1.89 had a similarly high sensitivity (64%) but much lower specificity (34%) [21]. Sandobal et al. reported the thickness of the nail bed of over 2.0 mm as a cut-off value in psoriatic change diagnostics [20].

After the patients with Ps and PsA were grouped according to clinical manifestations of psoriatic changes in nails, the thickness of the nail plate, bed, and matrix was significantly greater in the onycholysis and hyperkeratosis-type changes (concomitant or occurring separately) than in the pitting-type changes (p=0.007, p=0.035, and p=0.023, respectively). In both groups of patients, morphological changes were assessed in nails by US examination in accordance with the classification proposed by Wortsman et al. Type I (focal hyperechoic of dorsal plate) was usually found in an examination of the affected nails in patients with psoriasis without arthritis. The ultrasound changes in the affected nails in patients with PsA usually involved loosening of the borders of the ventral plate (type II). Similar findings were reported by Sandobal et al.; in their study, type I was found in 16/20 of the Ps patients, whereas type II was found in 34/35 of the PsA patients, irrespective of the clinical manifestations of psoriatic nail involvement [20].

The features of enthesopathies of the digital extensor tendon in the DIP joint as determined in the US examination were present in 34% patients with psoriasis with nail involvement without the concomitant arthritis and in 52% of PsA patients. The features of enthesopathies were observed in joints of digits with nail involvement both in patients with Ps and with PsA (38% versus 66%, respectively). The literature provides reports on more frequent occurrence of enthesopathies in psoriasis without arthritis [22, 23], but few studies have been conducted so far of using US to assess the connection between psoriatic changes in nails with enthesopathy of the digital extensor tendon in the DIP joint. In a recently published paper by Acosta-Felquer et al., the frequency of enthesopathies in patients with psoriasis and PsA in a US examination of digits with nail involvement did not differ and was 61% and 60%, respectively [24]. In another study, Ash et al. described the relationship between the occurrence of psoriatic changes in nails and the intensity of enthesopathies in joints other than digital [25]. In another study, Castellanos-González et al. conducted US examinations in patients with Ps and found the presence of enthesopathy in 31% of patients with onycholysis. The frequency of changes in the digital extensor tendon or its attachment in the DIP joint of digits with nail involvement was nearly 83% [26].

Increased vascularisation may be one of the manifestations of an open or subclinical inflammation. As expected, increased vascularisation around the digital extensor tendon in the DIP joint, as assessed by the PD technique, was observed in the PsA patients more frequently than in those with Ps. An intense PD signal was also observed around the tendon in the DIP joints of digits with nail involvement in both groups of patients. Unlike in the study by Acquiter et al., no difference regarding the PD signal in DIP joints was observed in Ps patients between those with and without affected nails [27]. Sandobal et al. observed increased vascularisation in 106/350 DIP joints in PsA patients and only in 8/200 joints in Ps patients, irrespective of changes in nails [20]. Acosta-Felquer et al. found the frequency of a PD signal in the extensor tendon and the DIP joint in groups of Ps and PsA patients to be higher when nails were affected, but there was no difference between patients with and without arthritis [24].

A potential limitation of this study must be mentioned which results from the fact that present nail changes cannot be hidden in a US examination, which means that the study was not completely blinded.

## 7. Conclusions

The intensity of psoriatic changes in nails in Ps and PsA patients was associated with increased thickness of nail plates, bed, and matrix. The nail plate thickness increased with duration of the skin disease in all the examined patients. The thickness of the nail bed in PsA patients increased with the duration of arthritis and was correlated with the number of swollen joints. An increased PD signal in the nail bed area was observed in patients with arthritis more often than in Ps patients, regardless of any clinical nail changes. The findings of this study may indicate the association of an inflammation in the nail bed with PsA development. Apart from a direct assessment of the described morphological changes of nails, a US examination could prove useful in an assessment of intensity of a local inflammation as a prognostic factor for PsA development. However, it should be emphasized that the US requires special training and should be performed by a person experienced in ultrasound examinations. This may limit the availability of an ultrasound examination in everyday clinical practice. The practical use of such an assessment should be evaluated in further studies.

## Figures and Tables

**Table 1:** Age and clinical characteristic of the patients studied.

	Ps (n=38)	PsA (n=31)	Control (n=30)	p
male/female	20/18	15/16	14/16	
Age (years)	48.4± 9.1	49.7± 10.8	49.4 ± 12.7	ns
Ps duration (years)	17.8±10.9	19.3±6.6	-	ns
PsA duration (years)	-	7.8±7.1	-	-
DAS 28	-	3.3 ± 0.6	-	-
PASI	6.2±3.8	4.7±3.7	-	0.034
mNAPSI	21.9±14.9	20.3±15.2	-	ns
ESR	13.9±6.6	25.7±10.2	-	0.017
CRP (mg/dl)	2.7±2.4	9.4±4.1	-	<0.001
TJC	-	2.8±1.4	-	-
SJC	-	2.3±0.6	-	-
DMARD therapy	17/38	27/31	-	

Results are presented as numbers or mean values and standard deviations (SD).

Ps: psoriasis, PsA: psoriatic arthritis, DAS: disease activity score, PASI: psoriasis area severity index, mNAPSI: modified nail psoriasis severity index, ESR: erythrocyte sedimentation rate, CRP: C-reactive protein, TJC: tender joint count, SJC: swollen joint count, and DMARD: disease-modifying antirheumatic drug.

**Table 2:** US measurements of the fingers with psoriatic nail involvement compared to control.

	Ps (248/380)	PsA (168/310)	Control (n=298)	p
NP thickness (mm)	0.73± 0.14	0.72± 0.08	0.51 ± 0.12	<0.001
NB thickness (mm)	2.02±0.37	2.06±0.39	1.76 ± 0.22	0.003
Matrix thickness (mm)	1.96±0.36	2.01±0.24	1.88± 0.27	0.037
Tendon thickness (mm)	0.97±0.12	1.10±0.21	0.89±0.11	0.007

Results are presented as mean values and standard deviations (SD).

NP: nail plate; NB: nail bed;

**Table 3:** US measurements of the fingers without psoriatic nail involvement compared to control.

	Ps (132/380)	PsA (168/310)	Control (n=298)	p
NP thickness (mm)	0.59± 0.09	0.58± 0.07	0.51 ± 0.12	0.062
NB thickness (mm)	1.92±0.28	1.94±0.17	1.76 ± 0.22	0.027
Matrix thickness (mm)	1.90±0.14	1.93±0.19	1.88± 0.27	0.052
Tendon thickness (mm)	0.91±0.13	1.02±0.21	0.89±0.11^1^	0.036

Results are presented as mean values and standard deviations (SD).

NP: nail plate; NB: nail bed.

^1^p =0.133 Ps versus control.

**Table 4:** Wortsman classification of the psoriatic nails studied.

Wortsman classification	Ps (n=248)	PsA (n=168)
I	198	27
II	31	129
III	10	9
IV	-	3

## Data Availability

The datasets generated during and/or analysed during the current study are available from the corresponding author on reasonable request.
